# Supplementary material for: High-resolution modeling and projection of heat-related mortality in Germany under climate change
Source: Commun Med (Lond). 2024 Oct 21;4:206. doi: 10.1038/s43856-024-00643-3 (PMC11494177; doi:10.1038/s43856-024-00643-3)
Supplement: Supplementary file 1 — Supplementary Information [file 43856_2024_643_MOESM1_ESM.pdf]

Supplementary Figure 1

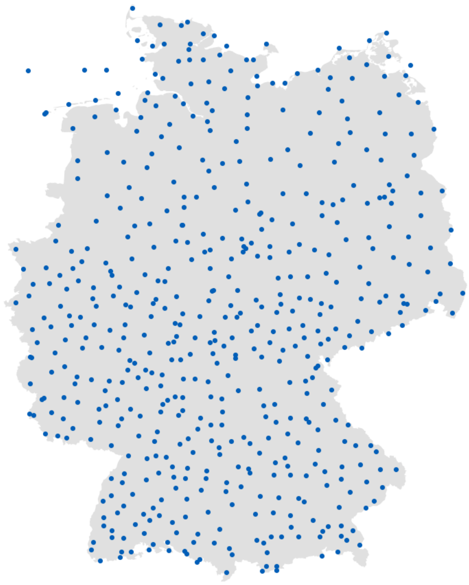

Supplementary Figure 1: Geographical distribution of the selected 537 weather stations.

Supplementary Figure 2

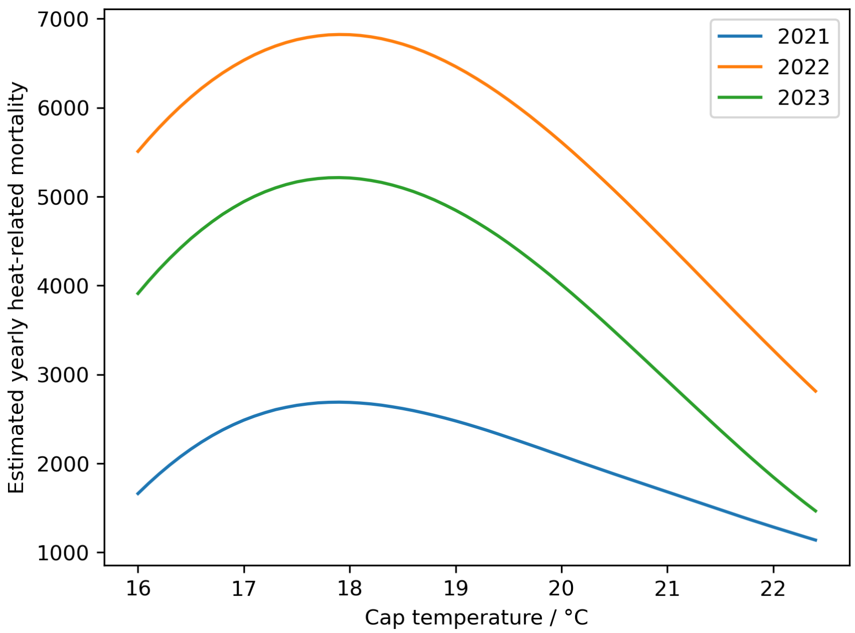

Supplementary Figure 2: Impact of selected cap temperature on heat-related mortality estimation for the years 2021-2023. The minimum mortality temperature lies at 18°C, resulting in the highest estimation of heat-related mortality.

Supplementary Figure 3

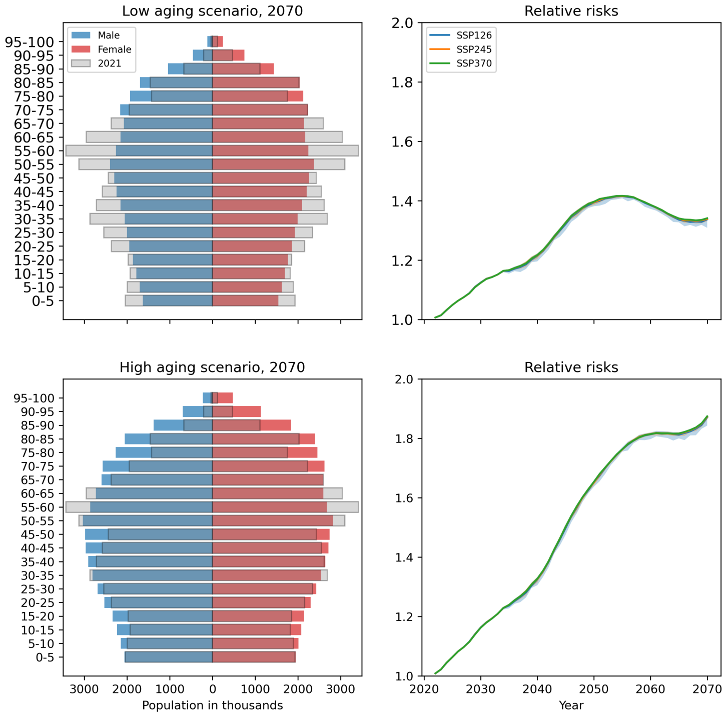

Supplementary Figure 3: Left: Population structure in 2070 for the low aging scenario (top panel, slight increase in life expectancy and low net migration, variant 13) and high aging scenario (bottom panel, sharp increase in life expectancy and high net migration, variant 20).[4] The grey bar represents the population in 2021. Right: Relative changes in population-level heat-related mortality risk compared to the static population structure in 2021 as in Fig. 3 of the main text.

Supplementary Figure 4

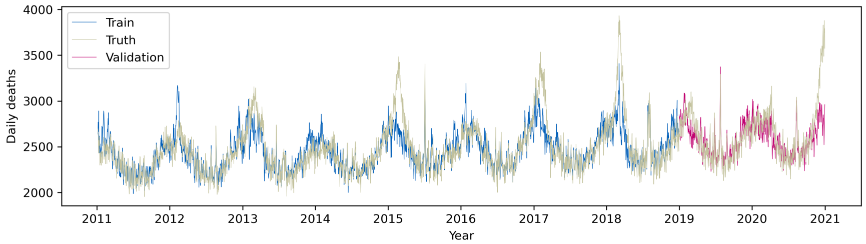

Supplementary Figure 4: Registered and estimated daily mortality in Germany with the best model. The temperature data in 2013 was replaced with the interpolated DWD data.

Supplementary Figure 5

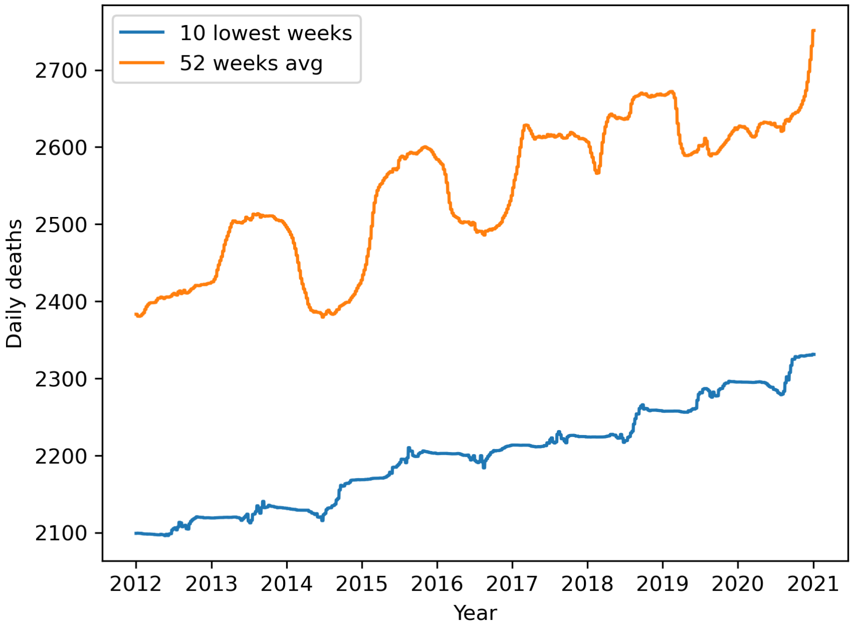

Supplementary Figure 5: Estimated mortality baseline with two different methods.

Supplementary Figure 6

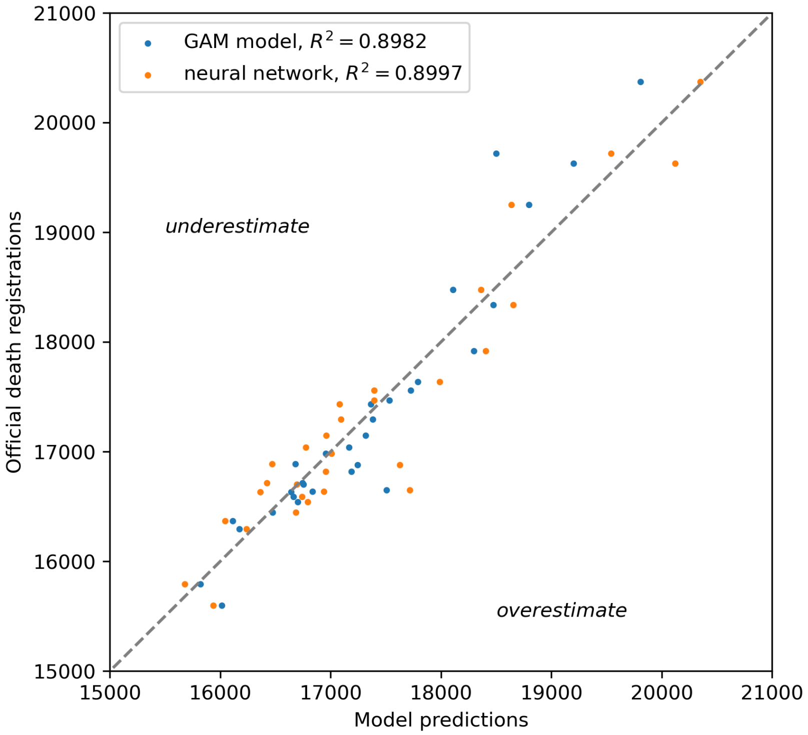

Supplementary Figure 6: Comparison of weekly estimated mortality with registered death cases for weeks with average temperatures over 20°C.

Supplementary Table 1

Supplementary Table 1: Estimated heat-related mortality with different temperature aggregation level. Italic text in parentheses represents the difference in heat-related mortality estimation compared to district-level temperature data.

	our model			RKI-report*
	District	State	Germany	
2014	2000	1700 *(-300)*	1100 *(-900)*	1300
2015	7800	6500 *(-1300)*	5200 *(-2600)*	5800
2016	2400	2100 *(-300)*	1500 *(-900)*	1700
2017	1800	1400 *(-400)*	800 *(-1000)*	1300
2018	9900	8600 *(-1300)*	6800 *(-3100)*	8300
2019	7700	6800 *(-900)*	5300 *(-2400)*	6900
2020	4800	4100 *(-700)*	2900 *(-1900)*	3600
2021	2100	1900 *(-200)*	1300 *(-800)*	1900
2022	5600	4900 *(-700)*	3500 *(-2100)*	4500
2023	4000	3500 *(-500)*	2800 *(-1200)*	3200

The models were trained using the average temperature data from Helmholtz Munich aggregated on district level with the average of the 10 lowest weeks in the previous year as mortality baseline. The reported results are averages derived from 20 ensemble members. The correction of day-of-the-week was applied. Due to COVID-19, a reliable baseline for mortality rates could not be calculated for 2021-2023; instead, the baseline mortality of the last week in 2020 was used.
* In RKI-reports Germany is grouped into 3 large regions.[1]

Supplementary Table 2

Supplementary Table 2: Impact of model structure and lag days

RMSE(R^2)	ReLU		Exponential	
lag days	train	validation	train	validation
no lag	145.77 *(0.463)*	174.42 *(0.252)*	149.43 *(0.436)*	193.10 *(0.084)*
1 day	114.59 *(0.668)*	130.74 *(0.580)*	123.85 *(0.612)*	158.27 *(0.384)*
2 days	104.90 *(0.722)*	124.05 *(0.622)*	110.60 *(0.691)*	133.13 *(0.564)*
3 days	96.52 *(0.765)*	132.30 *(0.570)*	110.06 *(0.694)*	121.56 *(0.637)*
4 days	93.69 *(0.778)*	144.72 *(0.485)*	111.50 *(0.686)*	111.21 *(0.696)*
5 days	88.94 *(0.800)*	137.57 *(0.535)*	112.99 *(0.677)*	107.14 *(0.718)*
6 days	86.77 *(0.810)*	127.06 *(0.603)*	115.24 *(0.664)*	107.47 *(0.716)*
7 days	81.59 *(0.832)*	124.16 *(0.621)*	113.97 *(0.672)*	106.21 *(0.723)*
8 days	74.83 *(0.858)*	119.00 *(0.652)*	110.22 *(0.693)*	108.23 *(0.712)*
9 days	72.71 *(0.866)*	117.31 *(0.662)*	107.41 *(0.708)*	112.17 *(0.691)*

The models were trained using the average temperature data from Helmholtz Munich, with the average mortality from the previous year serving as the baseline. Day-of-the-week correction was not included in the training. The reported results are averages derived from 20 ensemble members. Root mean square error(RMSE) and coefficients of determination(R^2) were calculated for hot days, defined as days with a Germany-wide average temperature exceeding 20°C. Data from the year 2013 was excluded due to irregularities.

Supplementary Table 3

Supplementary Table 3: Variance within ensemble members

RMSE	ReLU		Exponential	
5 lag days	train	validation	train	validation
error of mean	88.88	136.88	112.94	107.43
mean of error	95.78± 3.25	147.61±8.10	113.38±0.88	108.18±1.65
max of error	103.69	167.06	116.07	113.06

The models were trained using the average temperature data from Helmholtz Munich, with the average mortality from the previous year serving as the baseline. Day-of-the-week correction was not included in the training. The reported results are averages derived from 100 ensemble members. Root mean square errors were calculated for hot days, defined as days with a Germany-wide average temperature exceeding 20°C. We employed two different methods to calculate the RMSE. In the 'error of the mean,' we calculated the average result of all ensemble members and compared it to registered deaths. In the 'mean of error,' we compared the result of each individual ensemble member to registered deaths to obtain the RMSE. We then calculated the corresponding mean and standard error. Additionally, we listed the maximum error among all ensemble members as a reference. Data from the year 2013 was excluded due to irregularities.

Supplementary Table 4

Supplementary Table 4: Impact of baseline selection

RMSE(R^2)	Exponential	
5 lag days	train	validation
constant	115.92 (*0.660*)	125.36 (*0.614*)
52 weeks avg	112.99 (*0.677*)	107.14 (*0.718*)
10 lowest weeks	96.91 (*0.763*)	98.28 (*0.763*)

The models were trained using the average temperature data from Helmholtz Munich with different baselines. Day-of-the-week correction was not included in the training. The reported results are averages derived from 20 ensemble members. Root mean square error(RMSE) and coefficients of determination(R^2) were calculated for hot days, defined as days with a Germany-wide average temperature exceeding 20°C. Data from the year 2013 was excluded due to irregularities.

Supplementary Table 5

Supplementary Table 5: Impact of temperature dataset

RMSE	Exponential	
5 lag days	train	validation
1×1 km, max	117.40 *(0.626)*	117.68 *(0.642)*
1×1 km, mean	96.91 *(0.763)*	98.28 *(0.763)*
1×1 km, min	110.06 *(0.683)*	109.07 *(0.714)*
1×1 km, 3 channels	94.83 *(0.773)*	97.59 *(0.766)*
CERRA, 6-hourly	97.57 *(0.757)*	133.48 *(0.567)*

The models were trained with the average of the 10 lowest weeks in the previous year as mortality baseline. Day-of-the-week correction was not included in the training. The reported results are averages derived from 20 ensemble members. Root mean square error(RMSE) and coefficients of determination(R^2) were calculated for hot days, defined as days with a Germany-wide average temperature exceeding 20°C. Data from the year 2013 was excluded due to irregularities.

Supplementary Table 6

Supplementary Table 6: Impact of day-of-the-week correction

RMSE	Exponential	
5 lag days	train	validation
no correction	96.91 *(0.763)*	98.28 *(0.763)*
with correction	91.43 *(0.789)*	83.85 *(0.827)*

The models were trained using the average temperature data from Helmholtz Munich with the average of the 10 lowest weeks in the previous year as mortality baseline. The reported results are averages derived from 20 ensemble members. Root mean square errors were calculated for hot days, defined as days with a Germany-wide average temperature exceeding 20°C. Data from the year 2013 was excluded due to irregularities.

Supplementary Table 7

Supplementary Table 7: Performance of best model

RMSE	train	validation
hot days	91.4	83.9
all days	171.1	199.6

The models were trained using the average temperature data from Helmholtz Munich with the average of the 10 lowest weeks in the previous year as mortality baseline. The reported results are averages derived from 20 ensemble members. Root mean square errors were calculated for hot days (days with a Germany-wide average temperature exceeding 20°C) and all days. The correction of day-of-the-week was applied. Data from the year 2013 was excluded due to irregularities.

Supplementary Table 8

Supplementary Table 8: Comparison of heat-related mortality of the whole year and top 10 days

	whole year	top 10 days	percentage	dates
2014	2000	1500	76%	09-12 Jun, 18-23 Jul
2015	7800	4600	59%	03-07 Jul, 07-10 Aug,14 Aug
2016	2400	1500	62%	24-25 Jun, 20-22 Jul, 26-29 Aug, 14 Sep
2017	1800	1000	54%	29-30 May, 21-24 Jun, 08-09 Jul, 02-03 Aug
2018	9900	5600	57%	27-28 Jul, 31 Jul, 01-05 Aug, 07-08 Aug
2019	7700	4700	60%	26-27 Jun, 30 Jun, 24-29 Jul, 28 Aug
2020	4800	3400	72%	08-15 Aug, 21-22 Aug
2021	2100	1800	88%	17-22 Jun, 28-29 Jun, 14-15 Aug
2022	5600	2500	44%	19 Jun, 20-23 Jul, 04-05 Aug, 14-15 Aug, 17 Aug
2023	4000	2000	50%	22 Jun, 09-12 Jul, 19-23 Aug

The models used were the same as those presented in Extended Data Table 7. Due to COVID-19, a reliable baseline for mortality rates couldn't be calculated for 2021-2023; instead, the baseline mortality of the last week in 2020 was used. We identified the top 10 days with the highest estimated heat-related deaths. These days may not necessarily be the hottest 10 days due to the lag effect in the temperature-mortality response curve.

Supplementary Discussion

The daily temperature data used in our model allows for a closer examination of the temperature's influence on mortality. In addition, we tested the effects of maximum, minimum and average temperatures on mortality. While the model using average temperature demonstrated the best performance, the model using minimum temperature outperformed the one using maximum temperature. These results confirmed the findings of previous studies, which suggest that high night temperatures during a heatwave are more fatal to vulnerable populations.[2]

Additionally, our model enabled further inspection into the factors impacting the model's accuracy. Shkolnikov et al. suggested different methods for estimating the mortality baseline.[3] The suggested baseline estimated a higher number of excess deaths compared to the traditional method. However, no validation was provided in the paper. With our model, we confirmed that the average mortality from the 10 lowest weeks in the last year is a closer estimation of the mortality rate baseline compared to the traditional methods. The main reason for the differences is the inclusion of seasonal influenza impact in yearly averages. The impact leads to strong variation of the baseline and adversely affects the performance of the model. These results further indicated that the conventional excess death estimation underestimated the potentially avoidable losses, especially in winter. It is important to mention that COVID-19 had significant impact on mortality baseline estimation. Typically, the 10 weeks with the lowest mortality are in the summer months. However, in 2022, mortality during the summer was unusually high due to the impact of COVID-19, making it unsuitable for the baseline estimation.

The performance improvement with the correction factors for each day of the week highlighted irregularities in the registration data. Death registrations may experience delays during weekends or holidays. Thus, it is challenging to separate the lag effect due to the delay of the registration from the lag effect of the temperature on mortality. As we had no national-wide hospitalization data available, we decided to rely on the data from the official death registration and used different methods to validate our model. Further studies of the relationship between the mortality and hospitalization during heatwaves will help to guide the preparation of healthcare system of future heatwaves.

Supplementary References

[1] Matthias an der Heiden, Christoph Winklmayr, Sabine Buchien, Marion Schranz, RKI-Geschäftsstelle für Klimawandel & Gesundheit, Michael Diercke, and Viviane Bremer. Wochenbericht zur hitzebedingten mortalität kw 38/2023 vom 05.10.2023. Robert Koch-Institut Report, 2023.

[2] Karine Laaidi, Abdelkrim Zeghnoun, Bénédicte Dousset, Philippe Bretin, Stéphanie Vandentorren, Emmanuel Giraudet, and Pascal Beaudeau. The impact of heat islands on mortality in paris during the august 2003 heat wave. *Environmental health perspectives*, 120(2):254–259, 2012.

[3] Vladimir M Shkolnikov, Ilya Klimkin, Martin McKee, Dmitri A Jdanov, Ainhoa Alustiza-Galarza, László Németh, Sergey A Timonin, Marília R Nepomuceno, Evgeny M Andreev, and David A Leon. What should be the baseline when calculating excess mortality? new approaches suggest that we have underestimated the impact of the covid-19 pandemic and previous winter peaks. *SSM-population health*, 18:101118, 2022.

[4] Statistisches Bundesamt. Künftige Bevölkerungsentwicklung in Deutschland, 2024. Accessed: 2024-06-13.
