# Supplementary material for: High-resolution modeling and projection of heat-related mortality in Germany under climate change
Source: Commun Med (Lond). 2024 Oct 21;4:206. doi: 10.1038/s43856-024-00643-3 (PMC11494177; doi:10.1038/s43856-024-00643-3)
Supplement: Supplementary file 2 — Reporting Summary [file 43856_2024_643_MOESM2_ESM.pdf]

nature portfolio

Corresponding author(s): Junyu Wang

Last updated by author(s): Aug 23, 2024

Reporting Summary

Nature Portfolio wishes to improve the reproducibility of the work that we publish. This form provides structure for consistency and transparency in reporting. For further information on Nature Portfolio policies, see our Editorial Policies and the Editorial Policy Checklist.

Statistics

For all statistical analyses, confirm that the following items are present in the figure legend, table legend, main text, or Methods section.

n/a	Confirmed	
☒	☐	The exact sample size (*n*) for each experimental group/condition, given as a discrete number and unit of measurement
☒	☐	A statement on whether measurements were taken from distinct samples or whether the same sample was measured repeatedly
☒	☐	The statistical test(s) used AND whether they are one- or two-sided<br>*Only common tests should be described solely by name; describe more complex techniques in the Methods section.*
☒	☐	A description of all covariates tested
☒	☐	A description of any assumptions or corrections, such as tests of normality and adjustment for multiple comparisons
☒	☐	A full description of the statistical parameters including central tendency (e.g. means) or other basic estimates (e.g. regression coefficient) AND variation (e.g. standard deviation) or associated estimates of uncertainty (e.g. confidence intervals)
☒	☐	For null hypothesis testing, the test statistic (e.g. *F*, *t*, *r*) with confidence intervals, effect sizes, degrees of freedom and *P* value noted<br>*Give P values as exact values whenever suitable.*
☒	☐	For Bayesian analysis, information on the choice of priors and Markov chain Monte Carlo settings
☒	☐	For hierarchical and complex designs, identification of the appropriate level for tests and full reporting of outcomes
☒	☐	Estimates of effect sizes (e.g. Cohen's *d*, Pearson's *r*), indicating how they were calculated

Our web collection on statistics for biologists contains articles on many of the points above.

Software and code

Policy information about availability of computer code

Data collection	No software was used to collect the data.
Data analysis	NumPy(1.26.3), Pandas(2.1.4), Pytorch(2.0.0), Lightning(2.0.9) are used to analyze the data

For manuscripts utilizing custom algorithms or software that are central to the research but not yet described in published literature, software must be made available to editors and reviewers. We strongly encourage code deposition in a community repository (e.g. GitHub). See the Nature Portfolio guidelines for submitting code & software for further information.

Data

Policy information about availability of data

All manuscripts must include a data availability statement. This statement should provide the following information, where applicable:

- Accession codes, unique identifiers, or web links for publicly available datasets
- A description of any restrictions on data availability
- For clinical datasets or third party data, please ensure that the statement adheres to our policy

The following data were used to train the model.

Temperature
- [CERRA reanalysis data](https://cds.climate.copernicus.eu/cdsapp#!/dataset/reanalysis-cerra-single-levels?tab=overview) (Copernicus, open data)

- [1x1 km daily temperature data](https://doi.org/10.1016/j.envres.2022.115062) (Helmholz Munich, non-open data)
- [Deutscher Wetterdienst(DWD) station data](https://www.dwd.de/DE/klimaumwelt/cdc/cdc_node.html) (Climate Data Center, open data)
- [ERA5 daily statistics calculator](https://cds.climate.copernicus.eu/cdsapp#!/software/app-c3s-daily-era5-statistics?tab=overview) (Copernicus, open data)
- [Climate projection](https://aims2.llnl.gov/search) (EC-Earth3, open data)

[District level population](https://www.regionalstatistik.de/genesis/online?operation=statistic&levelindex=0&levelid=1705590504131&code=12411#abreadcrumb) (Statistische Ämter des Bundes und der Länder, open data)

[Death statistic](https://www.destatis.de/DE/Themen/Gesellschaft-Umwelt/Bevoelkerung/Sterbefaelle-Lebenserwartung/Tabellen/sonderauswertung-sterbefaelle.html) (DESTATIS, open data)

[Coordinate of each district](https://public.opendatasoft.com/explore/dataset/georef-germany-kreis/information/?disjunctive.lan_code&disjunctive.lan_name&disjunctive.krs_code&disjunctive.krs_name&disjunctive.krs_name_short&sort=year&location=6,51.32946,10.45403&basemap=jawg.light) (open data)

The population and death data was preprocessed and is avaiable in data folder. The district level temperature data from 2021 to 2023 (acquired with TempModel based on DWD data) is avaiable for testing.

The training data is not available due to the data agreement. Instead, we provide the trained parameters in params folder.

The climate projection data is not available due to the size.

Human research participants

Policy information about studies involving human research participants and Sex and Gender in Research.

Reporting on sex and gender	N/A
Population characteristics	N/A
Recruitment	N/A
Ethics oversight	N/A

Note that full information on the approval of the study protocol must also be provided in the manuscript.

Field-specific reporting

Please select the one below that is the best fit for your research. If you are not sure, read the appropriate sections before making your selection.

☐ Life sciences ☐ Behavioural & social sciences ☒ Ecological, evolutionary & environmental sciences

For a reference copy of the document with all sections, see nature.com/documents/nr-reporting-summary-flat.pdf

Ecological, evolutionary & environmental sciences study design

All studies must disclose on these points even when the disclosure is negative.

Study description	Use temperature to predict mortality and further estimate heat-related mortality.
Research sample	All German population and deaths.
Sampling strategy	N/A. sampling is not applicable since we used open source data.
Data collection	Open source and data from cooperation partner
Timing and spatial scale	Since 2011 because the statistic of population in Germany changed in 2011.
Data exclusions	No data excluded.
Reproducibility	Multiple models are trained to examine the relationship between temperature and mortality.
Randomization	N/A. Randomization is not applicable since we used all data to train and validate the model.
Blinding	N/A. Blinding is not applicable since all data are open sourced.

Did the study involve field work? ☐ Yes ☒ No

Reporting for specific materials, systems and methods

We require information from authors about some types of materials, experimental systems and methods used in many studies. Here, indicate whether each material, system or method listed is relevant to your study. If you are not sure if a list item applies to your research, read the appropriate section before selecting a response.

Materials & experimental systems

n/a	Involved in the study
☒	☐ Antibodies
☒	☐ Eukaryotic cell lines
☒	☐ Palaeontology and archaeology
☒	☐ Animals and other organisms
☒	☐ Clinical data
☒	☐ Dual use research of concern

Methods

n/a	Involved in the study
☒	☐ ChIP-seq
☒	☐ Flow cytometry
☒	☐ MRI-based neuroimaging
